# Closed-loop controller based on reference signal tracking for absence seizures

**DOI:** 10.1038/s41598-022-10803-x

**Published:** 2022-04-25

**Authors:** Hudong Zhang, Yuting Chen, Yan Xie, Yuan Chai

**Affiliations:** School of Mathematics and Physics, Shanghai University of Electric Power, Shanghai, 201306 China

**Keywords:** Epilepsy, Cognitive control

## Abstract

Absent epilepsy is a kind of refractory epilepsy, which is characterized by 2–4 Hz spike and wave discharges (SWDs) in electroencephalogram. Open-loop deep brain stimulation (DBS) targeting the thalamic reticular nucleus (TRN) is an effective method to treat absent epilepsy by eliminating SWDs in the brain. Compared with open-loop DBS, closed-loop DBS has been recognized by researchers for its advantages of significantly inhibiting seizures and having fewer side effects. Since traditional trial-and-error methods for adjusting closed-loop controller parameters are too dependent on the experience of doctors, in this paper we designed two proportional integral (PI) controllers based on the basal ganglia-cortical-thalamic model, whose PI parameters are calculated from the stability of the system. The two PI controllers can automatically adjust the frequency and amplitude of DBS respectively according to the change of the firing rate detected by substantia nigra pars reticulata (SNr). The parameters of the PI controller are calculated based on the Routh-Hurwitz stability criterion of a linear system which transformed by the original system using controlled auto-regressive (CAR) model and recursive least squares (RLS) method. Numerical simulation results show that both PI controllers significantly destroy the SWDs of the cerebral cortex and restore it to the other two normal discharge modes according to the different target firing rate, which supplies a promising brain stimulation strategy.

## Introduction

Absence epilepsy has been widely studied for its unique pathogenesis^1,2^, including the main frequency of 2–4 Hz spike and wave discharges (SWDs) in electroencephalogram (EEG)^3,4^, sudden and temporary loss of consciousness^5^, and widespread seizures in the adolescent years^6,7^. Previous studies have suggested that the thalamus plays a crucial role in epileptic seizures and that abnormal feedback from the thalamus to the cerebral cortex is responsible for the appearance of SWDs^8,9^. Subsequently, electrophysiological records in the cerebral cortex and thalamus of epileptic patients also demonstrated the corticothalamic system was associated with SWDs^10,11^, and the corresponding corticothalamic model was established^12,13^. The basal ganglia, as the information processing unit of the brain, directly or indirectly participates in the information transmission between the cortex and the thalamus^14,15^. Clinical studies have shown that damage to basal ganglia can lead to various brain disorders, including epilepsy^16^, cognitive impairment, and Parkinson's disease^17,18^. The basal ganglia are composed of subthalamic nucleus (STN), globus pallidus internal (GPi), globus pallidus external (GPe), and substantia nigra pars reticulata (SNr), of which the most important output nucleus is SNr neurons. Moreover, experimental studies on SNr in rodent models suggested that SNr plays an important role in the control of absence epilepsy^19,20^. Therefore, given that underlying mechanism of SNr in the brain is not clear, further research on SNr is necessary.

Slow oscillations of 0.1–0.2 Hz in the human brain are traveling waves that can trigger the thalamic spindles and periodically sweep across the cerebral cortex^21^. Spindles, which are associated with slow oscillations, can be observed in the electroencephalogram (EEG) in deep sleep^22^. Furthermore, experimental records showed that sleep spindles disappear when the transmission between thalamus reticular nucleus (TRN) and specific relay nucleus (SRN) is disrupted^23,24^. Therefore, researchers believe that TRN can act as a pacemaker of the spindles, and that the slow oscillation of different brain regions can also be regulated by TRN^25,26^. In recent years, more and more biophysical models have been established to study TRN. Fan et al. achieved the interconversion between spindles and SWDs in the cerebral cortex by applying deep brain stimulation (DBS) stimulation to TRN^27^. Wang et al. investigated the mechanism of external stimulation on TRN in the cortical thalamic model^28^. These studies have stimulated our enthusiasm to study the underlying mechanism of DBS stimulation on TRN.

There are many ways to treat epilepsy and the traditional methods are drug therapy or surgical removal^29,30^. Although these methods have a certain control effect on the absence epilepsy, the risks of surgery and the side effects of drugs should not be ignored^31,32^. DBS stimulation may be an alternative treatment for epilepsy in some patients with drug immunity^33^. DBS stimulation, which uses implanted electrodes to send electrical pulses to specific areas of the brain, has been shown to significantly reduce the onset of absence seizures^34,35^. However, when we build DBS stimulation, we need to consider many constraints such as battery life, stimulation intensity, and dynamic changes of the system^36,37^. In recent years, closed-loop DBS stimulation has received more attention than traditional open-loop DBS stimulation^38^. Closed-loop DBS is a control method that adaptively adjusts parameters and optimizes battery utilization^39^. The design of the closed-loop DBS stimulus controller is mainly about the selection of biomarkers reflecting the absence epileptic state and the selection of reference signals adapting to the dynamic changes of the system^40,41^. Specifically, when the epilepsy marker changes, which means the seizure, the reference signal triggers our preset value, and then the closed-loop controller automatically updates the DBS parameters to control the seizure^34^. From a dynamic point of view, the nervous system is multi-stable during epileptic seizures, and the closed-loop DBS controller serves to pull the cortical state back to the normal attraction basin^42,43^. At present, the closed-loop brain stimulation, vagus nerve stimulation and spinal cord stimulation applied in clinical practice basically depend on the feedback of therapeutic effect to adjust the stimulation parameters, which results in the control parameters being empirically selected^44,45^. The responsive neurostimulation, such as RNS System (*NeuroPace, Inc. USA*), can provide on-demand stimulation based on the detection of abnormal signals in the lesion, but RNS System has been used clinically for a short time and only treats patients with one or two foci of epilepsy^46^. Therefore, there are at least two limitations to traditional brain stimulation strategies. Firstly, the process of closed-loop control parameter adjustment relies too much on the experience of the physician. Secondly, the relationship between absent epilepsy and basal ganglia was neglected when the closed-loop control strategy was developed.

To break through these limitations, we designed two PI controllers, whose PI parameters are calculated from the stability of the system. PI controller successfully tracked the change of the firing rate of SNr, the main output nucleus of basal ganglia. The design of PI controller is based on approximating the basal ganglia-cortical-thalamic system into a linear system using controlled auto-regressive (CAR) model and recursive least squares (RLS) method, and then using Routh-Hurwitz stability criterion to calculate PI parameters. There are two commonly used system identification methods for CAR model, one is maximum likelihood method, and the other is RLS method. Because of its simplicity and practicality, the RLS method is often applied to nervous system models^47^. For example, in the study of Su et al., the algorithm combining CAR model and RLS was used to successfully track the dynamic beta oscillation activity in the basal ganglia^48^. In addition, proportional integral (PI) controller is widely used in the field of control engineering because of its robust performance and simple implementation. Moreover, PI controller also plays an important role in the field of neuropathic diseases^49–51^. Proportional feedback stimulation was applied to rat epileptic foci to control paroxysmal seizures^52^. Amplitude proportional with integral bias and derivative control were designed for Parkinson’s disease^53^.

The rest of the paper is organized as follows. In Section “Model and measure”, the absence seizures model was introduced, the relation of stimulus–response was established through CAR model and RLS method and the PI controller was designed according to the parameters obtained by Routh-Hurwitz stability criterion. In Section “Results”, the results of numerical simulation were illustrated. In Section “Conclusion and discussion”, the conclusion was given.

## Model and measure

In this section, we selected the absence epilepsy model composed of cerebral cortex, thalamus, and basal ganglion as the carrier of deep brain stimulation (DBS) stimulation and the experimental platform for the generation of absence epilepsy data^54,55^. Then, considering the importance of substantia nigra pars reticulata (SNr) in basal ganglia and its high correlation with other neurological diseases, we selected the mean firing rate of SNr as a reference signal, and identified the linear relationship between the DBS stimulus parameters and the firing rate of SNr. Finally, we constructed a closed-loop controller using a linear controlled auto-regressive (CAR) model and Routh-Hurwitz stability criterion to control absence epilepsy.

### Introduction of absence seizures model

**Figure 1:**
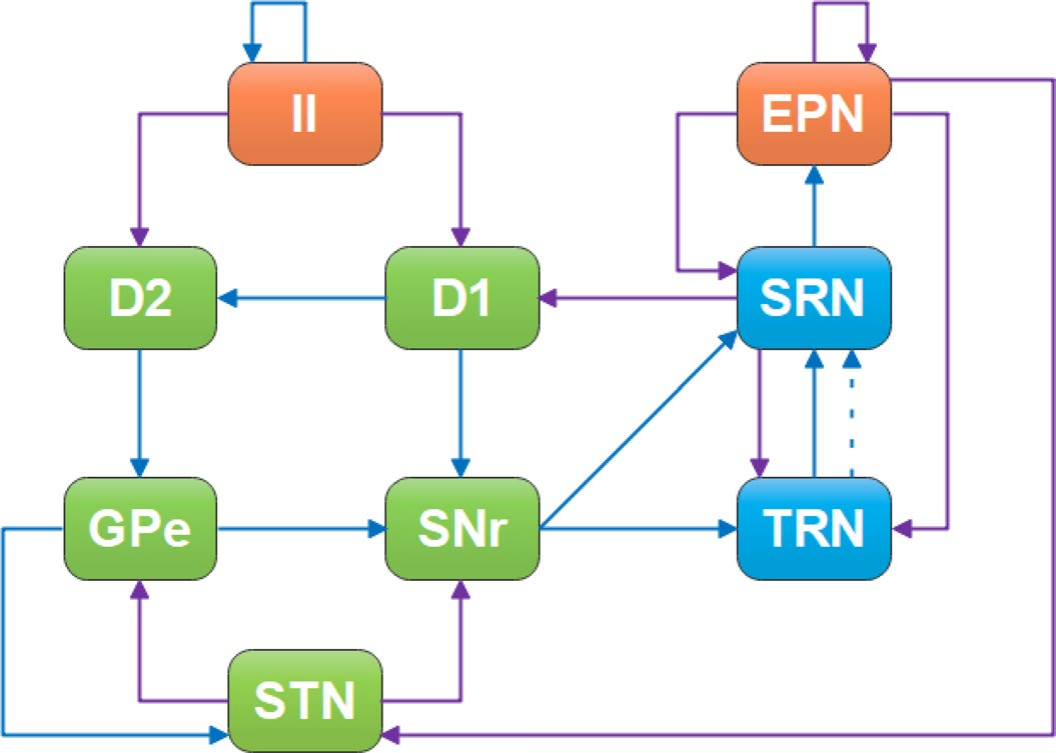
Structural diagram of a computational model for absent epilepsy. Nine neuron groups are shown as follows, *STN* subthalamic nucleus, *GPe* globus pallidus external, *SNr* substantia nigra pars reticulate, *D*_*2*_ striatal D_2_ neurons, *D*_*1*_ striatal D_1_ neurons, *SRN* specific relay nuclei, *TRN* thalamic reticular nucleus, *II* inhibitory interneurons, *EPN* excitatory pyramidal neurons. The excitatory projections are represented by purple arrow solid line. The blue lines represent inhibitory projections, where the solid and dot lines represent GABA_A_-mediated and GABA_B_-mediated, respectively.

The basal ganglia-cortical-thalamic network proposed by Chen et al. extends the basal ganglia based on the cortical-thalamic model^55,56^. Based on this model, we can successfully achieve the transformation of spike and wave discharges (SWDs) to spindle oscillations in the cerebral cortex. As shown in Fig. 1, the whole network model can be divided into three categories according to color, namely cerebral cortex (orange), thalamus (blue) and basal ganglia (green), each of which can be divided into different sub-modules. For example, the cerebral cortex is made up of two orange rectangles, called inhibitory interneurons (II) and excitatory pyramidal neurons (EPN). The different submodules are connected by projections: excitatory projections mediated by glutamate (purple arrow solid line), inhibitory projections mediated by GABA_A_ (blue arrow solid line), and inhibitory projections mediated by GABA_B_ (blue arrow dot line).

The biophysical model of absence epilepsy is shown as follows^55^:

1 $$ X^{\prime\prime}\left( t \right) = \alpha \beta \left( {C_{1} Y_{1} \left( t \right) - Y_{2} \left( t \right)} \right) - \left( {\alpha + \beta } \right)X^{\prime}\left( t \right), $$

2 $$ \phi^{\prime\prime}_{e} \left( t \right) = \gamma_{e}^{2} \left[ { - \phi_{e} \left( t \right) + F\left( {V_{EPN} } \right)} \right] - 2\gamma_{e} \phi^{\prime}_{e} \left( t \right), $$

3 $$ F\left[ {V_{i} \left( {r,t} \right)} \right] = \frac{{Q_{i}^{max} }}{{1 + \exp \left[ { - \frac{\pi }{\sqrt 3 }\frac{{\left( {V_{i} \left( {r,t} \right) - \theta_{i} } \right)}}{\sigma }} \right]}}, $$

where $$X\left( t \right) = \left[ {V_{EPN} \left( t \right),V_{{D_{1} }} \left( t \right),V_{{D_{2} }} \left( t \right),V_{SNr} \left( t \right),V_{GPe} \left( t \right),V_{STN} \left( t \right),V_{TRN} \left( t \right),V_{SRN} \left( t \right)} \right]^{T}$$,

4 $$ Y_{1} \left( t \right) = \left[ \begin{gathered} \phi_{e} ,F\left( {V_{EPN} } \right),F\left( {V_{{D_{1} }} } \right),F\left( {V_{{D_{2} }} } \right),F\left( {V_{SNr} } \right),F\left( {V_{GPe} } \right), \hfill \\ F\left( {V_{STN} } \right),F\left( {V_{TRN} } \right),F\left( {V_{TRN} \left( {t - \tau } \right)} \right),F\left( {V_{SRN} } \right) \hfill \\ \end{gathered} \right]^{T} , $$

5 $$ Y_{2} \left( t \right) = \left[ {V_{EPN} \left( t \right),V_{{D_{1} }} \left( t \right),V_{{D_{2} }} \left( t \right),V_{SNr} \left( t \right),V_{GPe} \left( t \right),V_{STN} \left( t \right),V_{TRN} \left( t \right),V_{SRN} \left( t \right) - \phi_{n} } \right]^{T} , $$

6 $$ C_{1} = \left[ {\begin{array}{*{20}c} {v_{EPNEPN} } & {v_{EPNII} } & 0 & 0 & 0 & 0 & 0 & {\left( {0,0} \right)} & {v_{EPNSRN} } \\ {v_{{D_{1} EPN}} } & 0 & {v_{{D_{1} D_{1} }} } & 0 & 0 & 0 & 0 & {\left( {0,0} \right)} & {v_{D1SRN} } \\ {v_{{D_{2} EPN}} } & 0 & 0 & {v_{{D_{2} D_{2} }} } & 0 & 0 & 0 & {\left( {0,0} \right)} & {v_{GPeSRN} } \\ 0 & 0 & {v_{{SNrD_{1} }} } & 0 & 0 & {v_{SNrGPe} } & {v_{SNrSTN} } & {\left( {0,0} \right)} & 0 \\ 0 & 0 & 0 & {v_{{GPeD_{2} }} } & 0 & {v_{GPeGPe} } & {v_{GPeSTN} } & {\left( {0,0} \right)} & 0 \\ {v_{STNEPN} } & 0 & 0 & 0 & 0 & {v_{STNGPe} } & 0 & {\left( {0,0} \right)} & 0 \\ {v_{TRNEPN} } & 0 & 0 & 0 & {v_{TRNSNr} } & 0 & 0 & {\left( {0,0} \right)} & {v_{TRNSRN} } \\ {v_{SRNEPN} } & 0 & 0 & 0 & {v_{SRNSNr} } & 0 & 0 & {\left( {v_{SRN,TRN}^{A} ,v_{SRN,TRN}^{B} } \right)} & 0 \\ \end{array} } \right], $$

where $\alpha$ is synaptodendritic decay time constant, and $\beta$ is synaptodendritic rise time constant. $\gamma_{e}$ denotes cortical damping rate, and $i \in \left[ {EPN,II,TRN,SRN,D_{1} ,D_{2} ,SNr,GPe,STN} \right]$ represents one of nine groups of neurons. $r$ indicates the spatial position, $\sigma$ represents the threshold variability of firing rate, $\theta_{i}$ represents the mean firing threshold, and $Q_{i}^{max}$ indicates the maximum firing rate. $\phi_{e}$ denotes cortical excitatory axonal field. $\tau$ and $\phi_{n}$ are represent the GABA_B_ delay and the constant nonspecific subthalamic input onto SRN. $v_{i,j}$ indicates coupling strength from $i$ to $j$,$i,j \in \left[ {EPN,II,TRN,SRN,D_{1} ,D_{2} ,SNr,GPe,STN} \right]$. The other parameters are shown in Table 1^55^.

**Table 1:** Parameter interpretation.

Parameter	Interpretation	Standard value ( mV s)
$v_{TRNSNr}$	Coupling strength SNr-TRN	0.035
$v_{SRNSNr}$	Coupling strength SNr-SRN	0.035
$v_{STNEPN}$	Coupling strength EPN-STN	0.1
$v_{SRNEPN}$	Coupling strength EPN-SRN	2.2
$v_{EPNSRN}$	Coupling strength SRN-EPN	1.8
$v_{STNGPe}$	Coupling strength GPe-STN	0.04
$v_{GPeSTN}$	Coupling strength STN-GPe	0.45
$v_{GPeGPe}$	Coupling strength GPe-GPe	0.075
$v_{{GPeD_{2} }}$	Coupling strength D_2_-GPe	0.3
$v_{SNrGPe}$	Coupling strength GPe-SNr	0.03
$v_{{SNrD_{1} }}$	Coupling strength D_1_-SNr	0.1
$v_{{D_{2} SRN}}$	Coupling strength D_2_-SRN	0.05
$v_{{D_{2} D_{2} }}$	Coupling strength D_2_-D_2_	0.3
$v_{{D_{2} EPN}}$	Coupling strength EPN-D_2_	0.7
$v_{{D_{1} SRN}}$	Coupling strength SRN-D_1_	0.1
$v_{{D_{1} D_{1} }}$	Coupling strength D_1_-D_1_	0.2
$v_{{D_{1} EPN}}$	Coupling strength EPN-D_1_	1
$v_{TRNSRN}$	Coupling strength SRN-TRN	0.5
$v_{TRNEPN}$	Coupling strength EPN-TRN	0.05
$v_{EPNII}$	Coupling strength II-EPN	1.8
$v_{EPNEPN}$	Coupling strength EPN-EPN	1

### Establish stimulus–response relation

**Figure 2:**
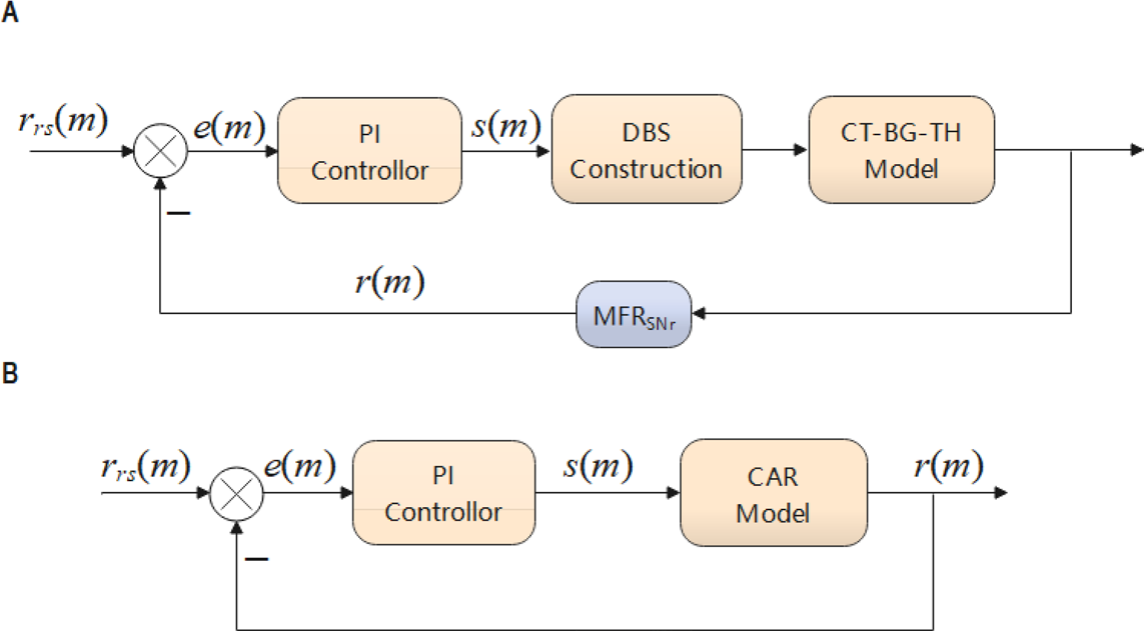
(**A**) The closed loop DBS controller. First, The mean firing rate of SNr is calculated from the absence epilepsy model and used as feedback signals $r\left( m \right).$ Then, the difference between the expected mean firing rate $r_{rs} \left( m \right)$ and $r\left( m \right)$ is used as input to the PI controller to calculate the frequency or amplitude of DBS stimulate $s\left( m \right)$. Finally, the DBS stimulus current input to the absence epilepsy model is constructed. (**B**) Linear system of closed-loop DBS controller.

The design diagram of the closed-loop DBS controller is illustrated in Fig. 2A. We selected the firing rate of SNr as the reference signal $r\left( m \right)$. Then, we input the difference between $r\left( m \right)$ and expected mean firing rate $r_{rs} \left( m \right)$ into the proportional integral (PI) controller to obtain the DBS stimulus parameter $s\left( m \right)$. Finally, we obtained DBS stimulation for the control of absence epilepsy. Since absence epilepsy is highly nonlinear, direct use of PI controller is not appropriate. Figure 2B shows the linear system of a closed-loop DBS controller, which is used to describe the linear relationship between DBS stimulus parameter and the mean firing rate of SNr. The CAR model is calculated as follows^57^:

7 $$ \left( {1 + a_{1} z^{ - 1} + a_{2} z^{ - 2} + \cdots + a_{{n_{a} }} z^{{ - n_{a} }} } \right)r\left( m \right) = \left( {b_{0} + b_{1} z^{ - 1} + b_{2} z^{ - 2} + \cdots b_{{n_{b} }} z^{{ - n_{b} }} } \right)s\left( m \right) + \varepsilon \left( m \right), $$

where *z*, $$r\left( m \right)$$, and $$s\left( m \right)$$ are the lag operator, output signal, and input signal, respectively. $$n_{a} ,$$$$\varepsilon \left( m \right)$$, and $$n_{b}$$ are the order of output, error, and the order of input, respectively.

**Figure 3:**
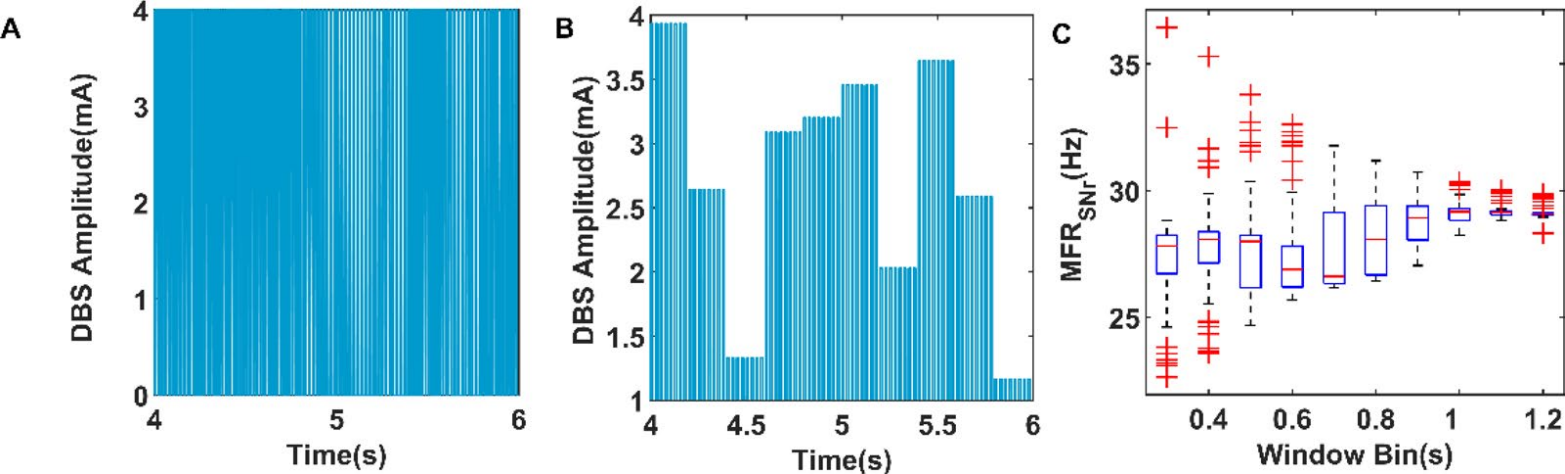
(**A**) The DBS current with different frequency. The duration of each current is 0.2 s, and the current frequency is between 20 and 100 Hz. (**B**) The DBS current with different amplitude. The duration of each current is 0.2 s, and the current amplitude is between 1 and 4 mA. (**C**) The box figure of mean firing rate of SNr plotted as a function of time window bin (60 trials), where the blue rectangle consists of the median, upper quartile and lower quartile, and the outliers are marked with red crosses. MFR_SNr_ represents mean firing rate of SNr.

As we know, the CAR model describes a linear relationship between stimulus and responses and relies heavily on input and output data. Therefore, to extract more information from the relationship between DBS parameters and mean firing rate of SNr, we let DBS parameters change randomly in a certain range. For example, Fig. 3A,B respectively show the discharge of DBS current during 4–6 s, and we change the frequency and amplitude of current respectively every 0.2 s. In addition, we also consider the influence of time window bin on the control accuracy of closed-loop DBS. As shown in Fig. 3C, we plotted the box diagram of mean firing rate of SNr as a function of the time window bin. The simulation results show that when the time window bin is 0.2 s, the mean firing rate of SNr measured each time is scattered, which means that the time window bin of 0.2 s is sensitive to the changes of the system.

The recursive least squares (RLS) method is used to estimate CAR model parameters $a_{1} ,a_{2} , \ldots ,a_{{n_{a} }}$ and $b_{1} ,b_{2} , \ldots ,b_{{n_{b} }}$. The least square form of the CAR model is shown as follows^57^:

8 $$ \begin{aligned} r\left( m \right) & = - a_{1} r\left( {m - 1} \right) - a_{2} r\left( {m - 2} \right) - \cdots - a_{{n_{a} }} r\left( {m - n_{a} } \right) \\ & \quad + b_{0} s\left( m \right) + b_{1} s\left( {m - 1} \right) + b_{2} s\left( {m - 2} \right) + \cdots + b_{{n_{b} }} s\left( {m - n_{b} } \right) + \varepsilon \left( m \right) \\ & = \varphi^{T} \left( m \right)\theta + \varepsilon \left( m \right), \\ \end{aligned} $$

where $\varphi \left( m \right) = \left[ { - r\left( {m - 1} \right), \cdots - r\left( {m - n_{a} } \right),s\left( m \right), \cdots s\left( {m - n_{b} } \right)} \right]^{T}$ is composed of past input and output data and current input data. $\theta = \left[ {a_{1} ,a_{2} , \cdots ,a_{na} ,b_{0} ,b_{1} , \cdots ,b_{nb} } \right]^{T}$ can be estimated by the following equation:

9 $$ \left\{ \begin{gathered} \mathop \theta \limits^{ \wedge } \left( m \right) = \mathop \theta \limits^{ \wedge } \left( {m - 1} \right) + K\left( m \right)\left[ {y\left( m \right) - \varphi^{T} \left( m \right)\mathop \theta \limits^{ \wedge } \left( {m - 1} \right)} \right] \hfill \\ K\left( m \right) = \frac{{P\left( {m - 1} \right)\varphi \left( m \right)}}{{1 + \varphi^{T} \left( m \right)P\left( {m - 1} \right)\varphi \left( m \right)}} \hfill \\ P\left( m \right) = \left[ {I - K\left( m \right)\varphi^{T} \left( m \right)} \right]P\left( {m - 1} \right) \hfill \\ \end{gathered} \right. $$

**Figure 4:**
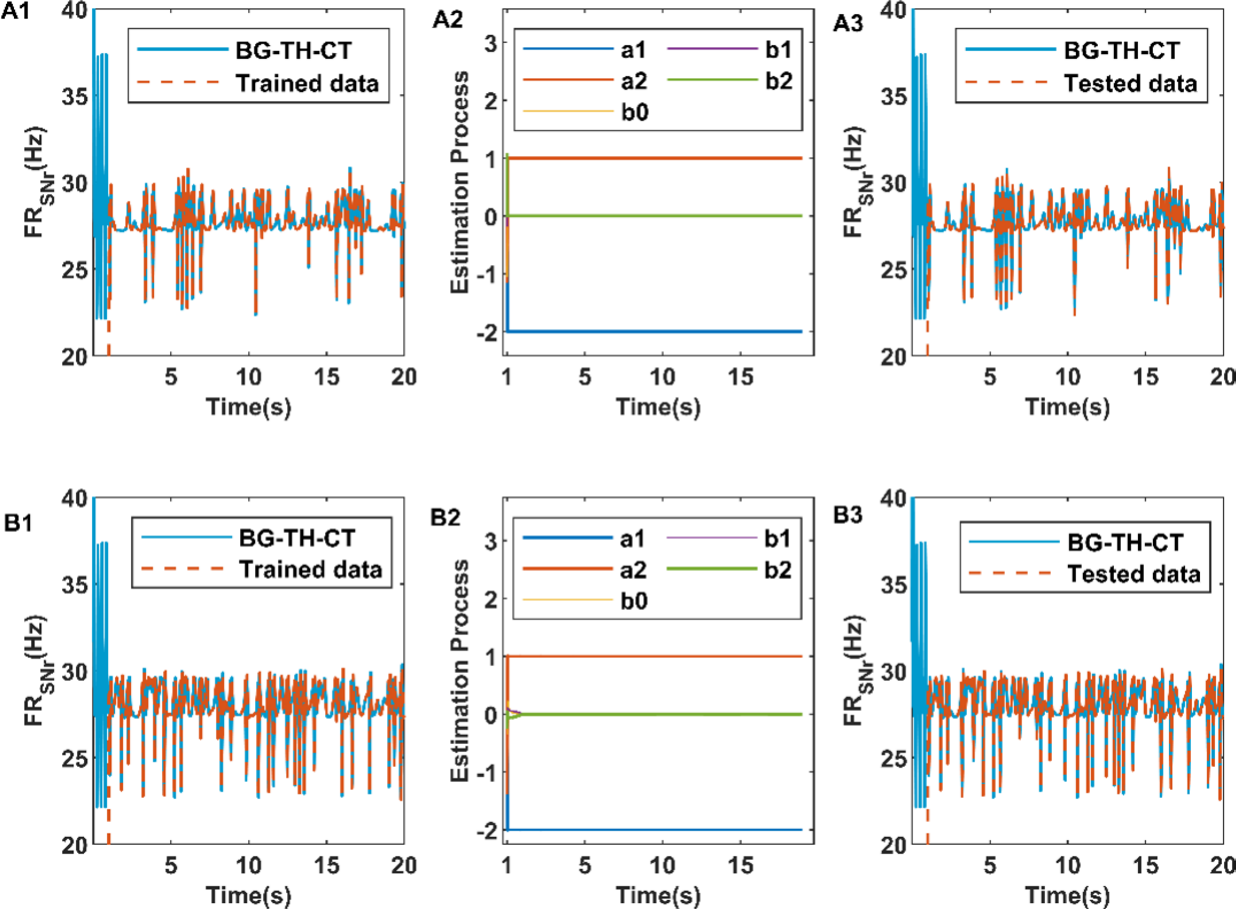
The performance of CAR models in model training (**A1**), parameter estimation (**A2**), and model testing (**A3**) when input of CAR model is DBS current frequency. The performance of CAR models in model training (**B1**), parameter estimation (**B2**), and model testing (**B3**) when input of CAR model is DBS current amplitude. In (**A1**,**B1**,**A3**,**B3**), the solid blue lines represent data from the basal ganglia-cortical-thalamic model. Dashed orange lines represent training data for CAR models in (**A1**) and (**B1**), and test data for CAR models in (**A3**) and (**B3**).

Figure 4 shows the performance of CAR model tracking dynamically changing firing rate of SNr when $n_{a} = 2$ and $n_{b} = 2$. We periodically change the DBS parameters from 1 s and start tracking the firing rate of SNr using the CAR model. When the input of CAR model is the frequency and amplitude of DBS current respectively, the corresponding performance of CAR model in model training is illustrated in Fig. 4A1,B1 respectively. Figure 4A2,B2 show the parameter estimation process of CAR model and the fitting of test data is shown in Fig. 4A3,B3 respectively. As we can see in Fig. 4, the CAR model performs well in training and testing, even though the order of the CAR model is relatively low. We should be more interested in the stability than the fitting accuracy of CAR model. Therefore, the CAR model in this paper is expressed as follows:

10 $$ r\left( m \right) = - a_{1} r\left( {m - 1} \right) - a_{2} r\left( {m - 2} \right) + b_{0} s\left( m \right) + b_{1} s\left( {m - 1} \right) + b_{2} s\left( {m - 2} \right). $$

### Design of closed-loop controller

The system replacing the absence epilepsy model with CAR model is shown in Fig. 2B and the structure of PI controller is as follows:

11 $$ s\left( m \right) = s\left( {m - 1} \right) + k_{p} \left[ {e\left( m \right) - e\left( {m - 1} \right)} \right] + k_{i} e\left( m \right), $$

where $k_{p}$ and $k_{i}$ are computed by the Routh-Hurwitz stability criterion, which is introduced by the following equations.

The forward transfer function of the system in Fig. 2B is as follows:

12 $$ \begin{gathered} G\left( z \right) = \frac{R\left( z \right)}{{E\left( z \right)}} = \frac{R\left( z \right)}{{S\left( z \right)}} \cdot \frac{S(z)}{{E(z)}} = \frac{{b_{0} z^{2} + b_{1} z + b_{2} }}{{z^{2} + a_{1} z + a_{2} }} \cdot \frac{{\left( {k_{p} + k_{i} } \right)z - k_{p} }}{z - 1} \hfill \\ \begin{array}{*{20}c} {} & {} \\ \end{array} \mathop {}\limits^{{}} = \frac{{b_{0} \left( {k_{p} + k_{i} } \right)z^{3} + \left[ {b_{1} \left( {k_{p} + k_{i} } \right) - b_{0} k_{p} } \right]z^{2} + [b_{2} \left( {k_{p} + k_{i} } \right) - b_{1} k_{p} ]z - b_{2} k_{p} }}{{z^{3} + \left( {a_{1} - 1} \right)z^{2} + \left( {a_{2} - a_{1} } \right)z - a_{2} }}. \hfill \\ \end{gathered} $$

The closed-loop transfer function is

13 $$ T\left( z \right) = \frac{G\left( z \right)}{{1 + G\left( z \right)}}. $$

The characteristic equation of this system is

14 $$ \begin{gathered} D\left( z \right) = 1 + G\left( z \right) = \left[ {1 + b_{0} \left( {k_{p} + k_{i} } \right)} \right]z^{3} + \left[ {\left( {a_{1} - 1} \right) + b_{1} \left( {k_{p} + k_{i} } \right) - b_{0} k_{p} } \right]z^{2} \hfill \\ \begin{array}{*{20}c} {} & {} \\ \end{array}_{{}} + \left[ {\left( {a_{2} - a_{1} } \right) + b{}_{2}\left( {k_{p} + k_{i} } \right) - b_{1} k_{p} } \right]z - b_{2} k_{p} - a_{2} = 0, \hfill \\ \end{gathered} $$

substituted $z$ with $w$, where $z = \frac{w + 1}{{w - 1}}$, we get

15 $$ D\left( w \right) = m_{3} \left( {\frac{w + 1}{{w - 1}}} \right)^{3} + m_{2} \left( {\frac{w + 1}{{w - 1}}} \right)^{2} + m_{1} \left( {\frac{w + 1}{{w - 1}}} \right) + m_{0} = 0, $$

where:

16 $$ \left\{ \begin{gathered} m_{3} = 1 + b_{0} \left( {k_{p} + k_{i} } \right), \hfill \\ m_{2} = \left( {a_{1} - 1} \right) + b_{1} \left( {k_{p} + k_{i} } \right) - b_{0} k_{p} , \hfill \\ m_{1} = \left( {a_{2} - a_{1} } \right) + b{}_{2}\left( {k_{p} + k_{i} } \right) - b_{1} k_{p} , \hfill \\ m_{0} = - b_{2} k_{p} - a_{2} . \hfill \\ \end{gathered} \right. $$

Then, multiplying both sides of equation by $\left( {w - 1} \right)^{3}$, we get

17 $$ \left( {w - 1} \right)^{3} D\left( w \right) = n_{3} w^{3} + n_{2} w^{2} + n_{1} w + n_{0} = 0, $$

where:

18 $$ \left[ \begin{gathered} n_{3} = m_{0} + m_{1} + m_{2} + m_{3} , \hfill \\ n_{2} = - 3m_{0} - m_{1} + m_{2} + 3m_{3} , \hfill \\ n_{1} = 3m_{0} - m_{1} - m_{2} + 3m_{3} , \hfill \\ n_{0} = - m_{0} + m_{1} - m_{2} + m_{3} . \hfill \\ \end{gathered} \right. $$

Based on the Routh-Hurwitz stability criterion, the stability of this system is equivalent to $n_{i} \left( {k_{p} ,k_{i} } \right) > 0\left( {i = 0,1,2,3} \right)$, and the corresponding subset of feasible solutions is $k_{p} \,\,{\text{and}}\,\,k_{i} > 0$.

Traditional closed-loop DBS designs mostly control the amplitude of DBS to adjust the intensity of DBS^58^. However, from the perspective of signal analysis, frequency modulation has stronger anti-interference ability than amplitude modulation. To sum up, we designed two types of closed-loop DBS controllers, one of which is based on frequency modulation (BoFM). We limit the frequency to 20–100 Hz, and the BoFM controller is shown as follows:

19 $$ s\left( m \right) = \left\{ {\begin{array}{*{20}c} {20} & {s\left( m \right) < 20} \\ {s\left( {m - 1} \right) + 5 * \left[ {e\left( m \right) - e\left( {m - 1} \right)} \right] + 0.3125 * e\left( m \right)} & {20 \le s\left( m \right) \le 100} \\ {100} & {s\left( m \right) > 100.} \\ \end{array} } \right. $$

The other is a closed-loop DBS controller based on amplitude modulation (BoAM). We limit the amplitude to 1–4 mA, and the BoAM controller is shown as follows:

20 $$ s\left( m \right) = \left\{ {\begin{array}{*{20}c} 1 & {s\left( m \right) < 1} \\ {s\left( {m - 1} \right) + 0.3 * \left[ {e\left( m \right) - e\left( {m - 1} \right)} \right] + 0.01875 * e\left( m \right)} & {1 \le s\left( m \right) \le 4} \\ 4 & {s\left( m \right) > 4.} \\ \end{array} } \right. $$

### Simulation analysis

In the paper, all the numerical calculations were conducted in the MATLAB R2019a (MathWorks, USA) simulation environment. The differential equation was solved by the standard fourth order Runge–Kutta method, with the temporal resolution of numerical integration is 0.05 ms. In the process of parameter training of CAR model, the optimal parameters can be obtained by running for sufficient time (> 15 s). The time series $\phi_{e}$ of cortical excitatory axon field was used to describe the cortical macro dynamics, the transient waveform which was unstable at the beginning was discarded to ensure that the time series used for analysis was obtained under the premise that the system was stable. The fast Fourier transform is used to estimate the dominant frequency of time series $\phi_{e}$.

## Results

### Dynamic change of firing rate in substantia nigra reticulum induced by open-loop deep brain stimulation

**Figure 5:**
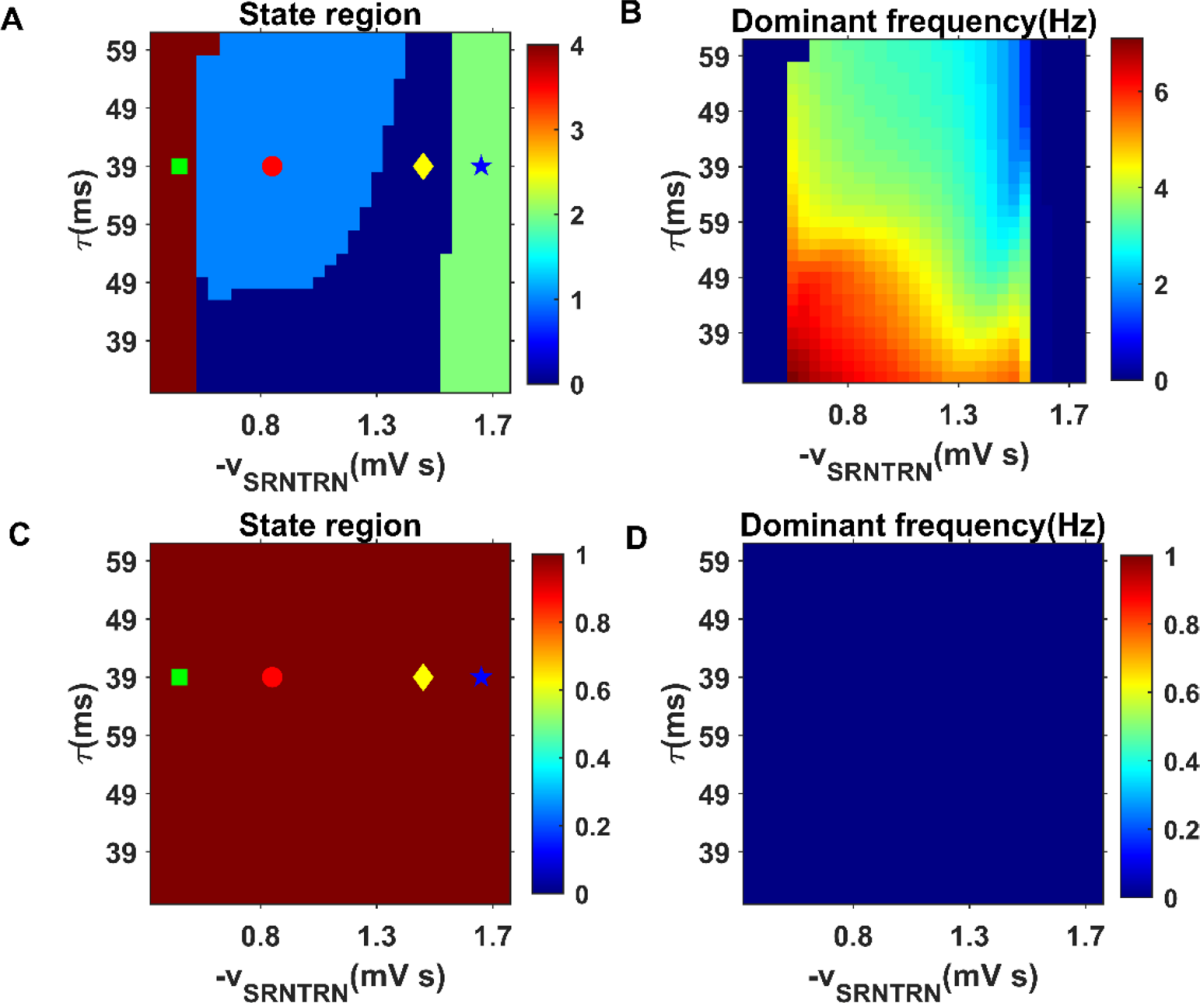
State (left) and dominant frequency (right) diagrams of the cerebral cortex in absence epilepsy model. The initial state and dominant frequency are shown in (**A**,**B**) respectively, while the state and dominant frequency with DBS stimulation is shown in (**C**,**D**) respectively. Different color regions in (**A**) represent different discharge states and are marked by different symbols: saturation state (green filled square), SWDs oscillation state (red filled circle), simple oscillation state (yellow filled diamond), low firing state (blue filled pentagram).

Our model successfully demonstrated the dynamic transition from spike and wave discharges (SWDs) firing to normal firing state by changing the inhibitory coupling strength $- v_{SRNTRN}$ and GABA_B_ delay $\tau$. State region diagrams and dominant frequency diagrams are used to describe the firing activity of the cerebral cortex as $- v_{SRNTRN}$ and $\tau$ change. Specifically, as $- v_{SRNTRN}$ changes, the cortical discharge activity can change from one state to another, and at the same time, the dominant frequency corresponding to the discharge activity changes, as shown in Fig. 5B,D. As shown in Fig. 5A, when the time delay is fixed at $\tau = 49$ ms, the firing state of the cerebral cortex first change from saturation state to SWDs state, and then from SWDs state to normal simple oscillation state with the increase of coupling strength $- v_{SRNTRN}$. Finally, the firing state of the cerebral cortex changes to low firing state due to the high inhibition of specific relay nuclei (SRN) by thalamic reticular nucleus (TRN). Moreover, when we add appropriate deep brain stimulation (DBS) to the TRN in absence epilepsy model, all firing regions of the cerebral cortex are inhibited. As shown in Fig. 5C, under the effect of DBS, the four firing states of the cerebral cortex are inhibited to the low firing state in the $\left( {\tau , - v_{SRNTRN} } \right)$ panel. Correspondingly, the main frequency of cortical electrical activity also decreases to 0 Hz in Fig. 5D.

**Figure 6:**
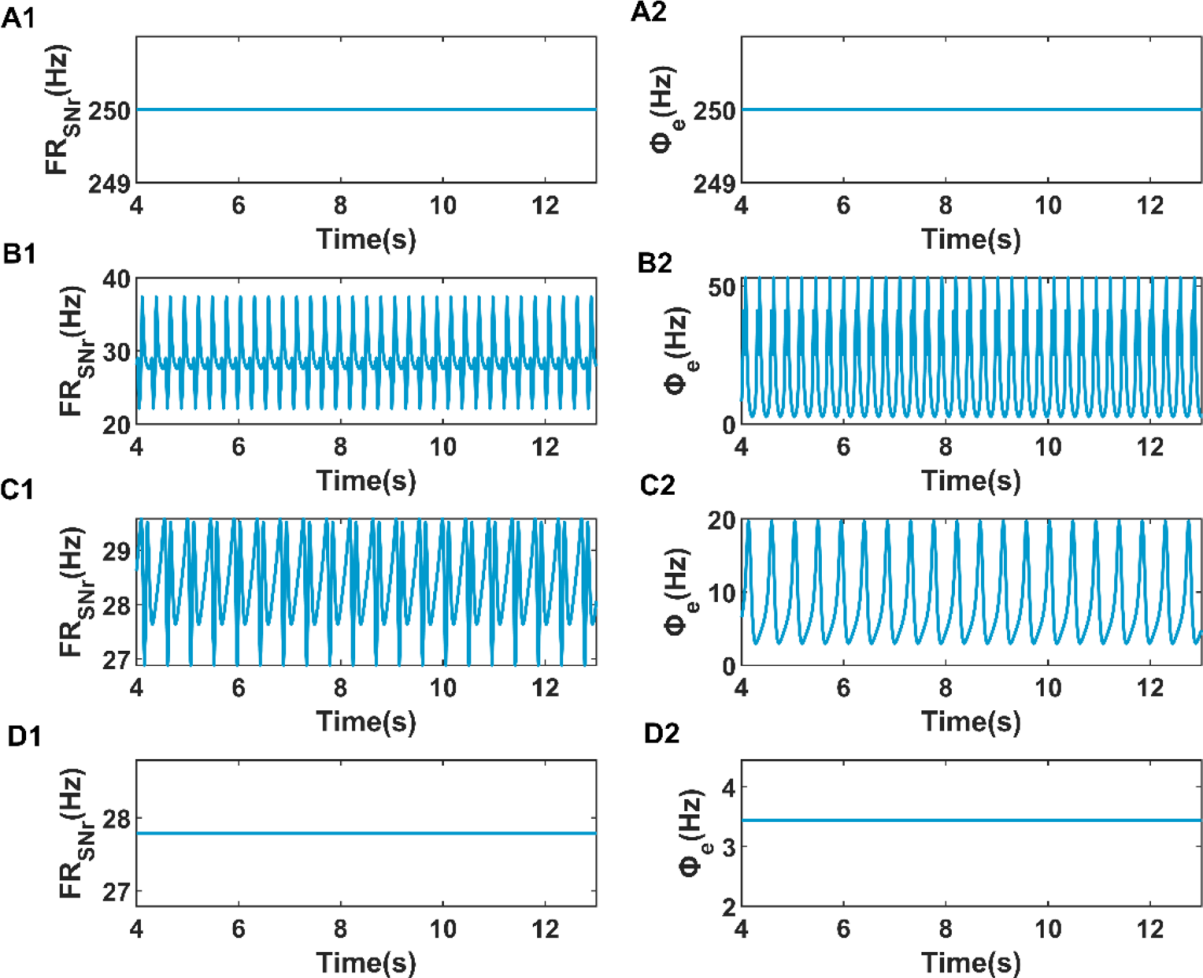
The four main kinds of firing activities (right) of the cerebral cortex and the corresponding firing rate (left) of the SNr. The firing activities of (**A2**,**B2**,**C2**,**D2**) correspond to saturation state, SWDs state, simple oscillation state and low firing state respectively and (**A1**,**B1**,**C1**,**D1**) show the firing rate of SNr in these four states.

As we can see, Fig. 6 clearly shows the four different firing states of the cerebral cortex and the corresponding firing rate of the substantia nigra pars reticulata (SNr). Figure 6A2–D2 respectively show the time series of cerebral cortex discharges in saturation state, SWDs state, simple oscillation state and low firing state, and the corresponding SNr firing rates are shown in Fig. 6A1–D1. Compared with the more stable firing rates such as saturation state and low firing state, the mean firing rates of SWDs oscillating state and simple oscillating state are constantly changing, which brings us difficulties in designing the closed-loop controller with mean firing rate as reference signal in the next section. For simplicity, we calculated the mean firing rate of four firing activity and find that the mean firing rate of SNr is arranged from high to low in the order of saturation state, SWDs oscillation state, simple oscillation state, and low firing state. Therefore, we chose the average value of firing rates of simple oscillation and low oscillation respectively as the expected firing rates of the closed-loop DBS controller, which are $r_{rs} = {27}{\text{.7884}}$ Hz and $r_{rs} = {28}{\text{.4144}}$ Hz, respectively.

**Figure 7:**
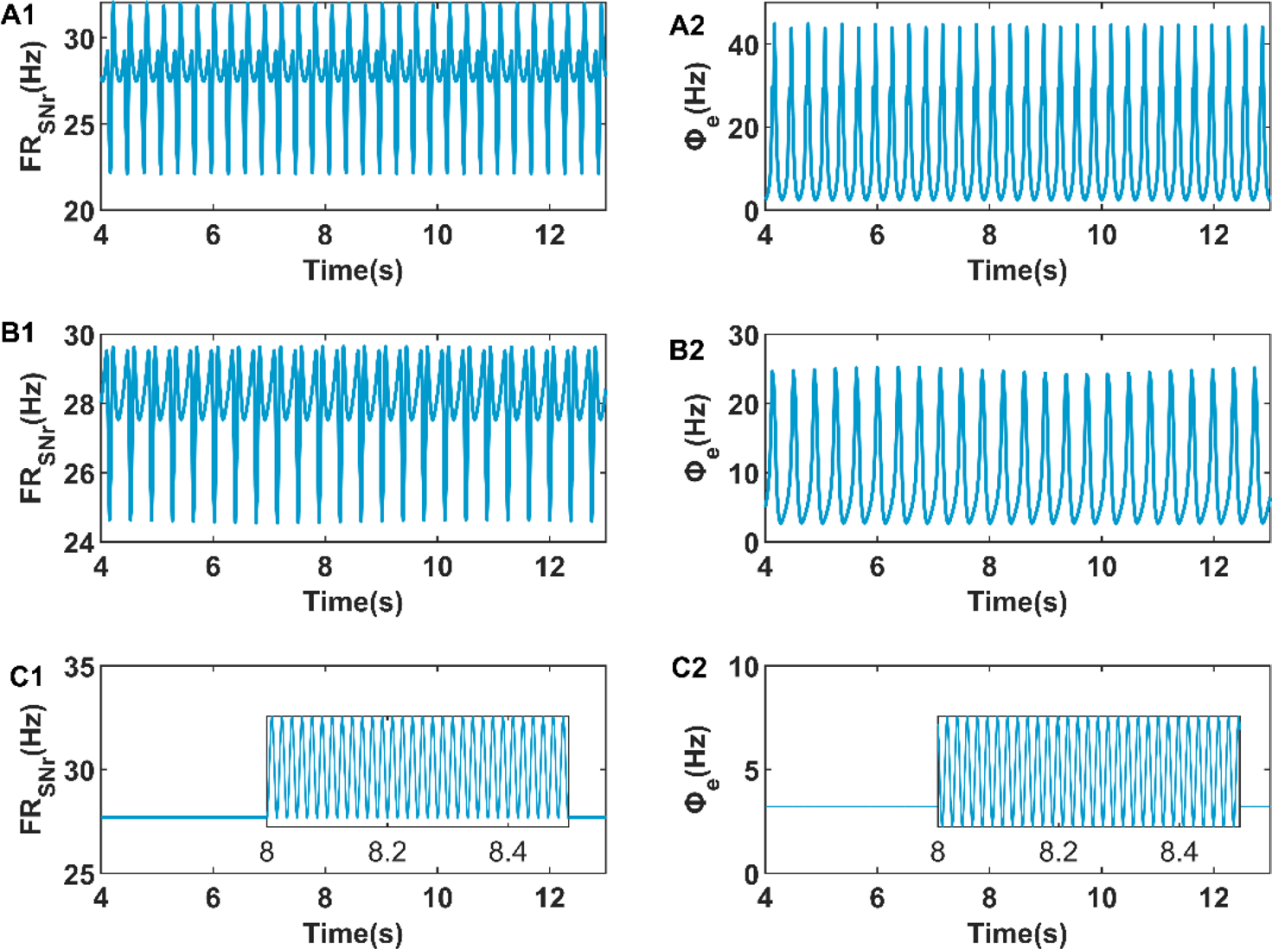
The firing rate of SNr when the frequency of DBS stimulation is 20 Hz (**A1**), 40 Hz (**B1**), and 60 Hz (**C1**). The firing activity of cerebral cortex when the frequency of DBS stimulation is 20 Hz (**A2**), 40 Hz (**B2**), and 60 Hz (**C2**).

**Figure 8:**
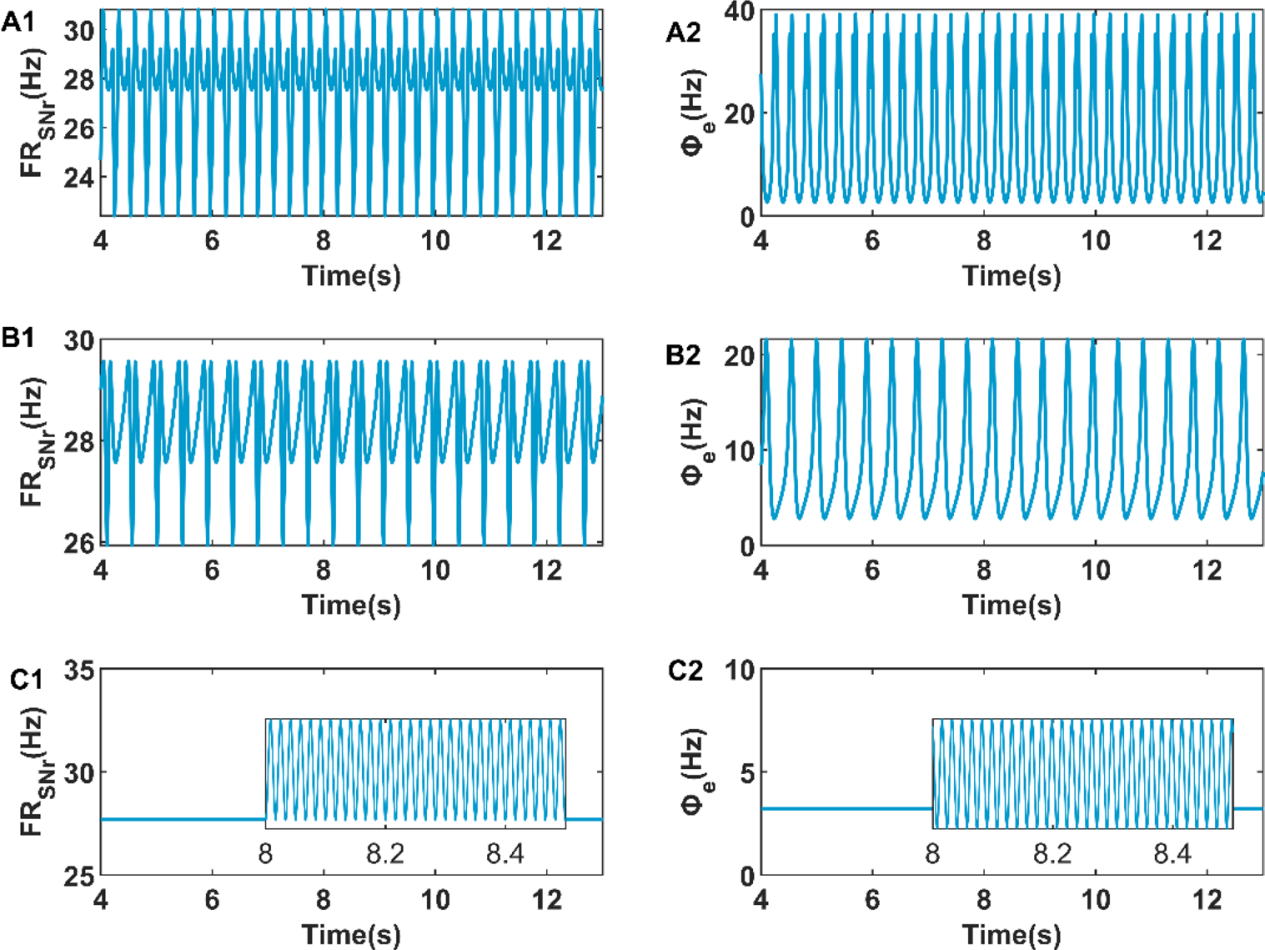
The firing rate of SNr when the amplitude of DBS stimulation is 1 mA (**A1**), 3 mA (**B1**), and 4 Hz (**C1**). The firing activity of cerebral cortex when the amplitude of DBS stimulation is 1 mA (**A2**), 3 mA (**B2**), and 4 mA (**C2**).

It has been known from Fig. 5C that DBS stimulation current can inhibit the firing activity of cerebral cortex, therefore we also want to research whether DBS stimulation current can also control the firing rate of SNr. For this purpose, we investigated the effects of frequency and amplitude of DBS stimulation on MFR of SNr. As shown in Fig. 7A1,A2,B1,B2, when the frequency of DBS stimulation increases from 20 to 40 Hz, the cortical firing activity changes from SWDs oscillation state to simple oscillation state, and the amplitude of the firing rate oscillation of SNr decreases. At the same time, we found a similar situation when the amplitude of DBS stimulus increased. As shown in Fig. 8A1,B1,A2,B2, as the amplitude of DBS stimulation increases from 1 to 3 mA, the amplitude of the firing rate of SNr decreases significantly, and the cortical firing activity changes from SWDs oscillation state to simple oscillation state. In addition, an interesting phenomenon is that we detected simple oscillations of 60 Hz with very low amplitude in the low firing state suppressed by DBS stimulation in Figs. 7C1,C2, 8C1,C2. This may be due to the introduction of artificial high-frequency electrical stimulation in the absence epilepsy model.

**Figure 9:**
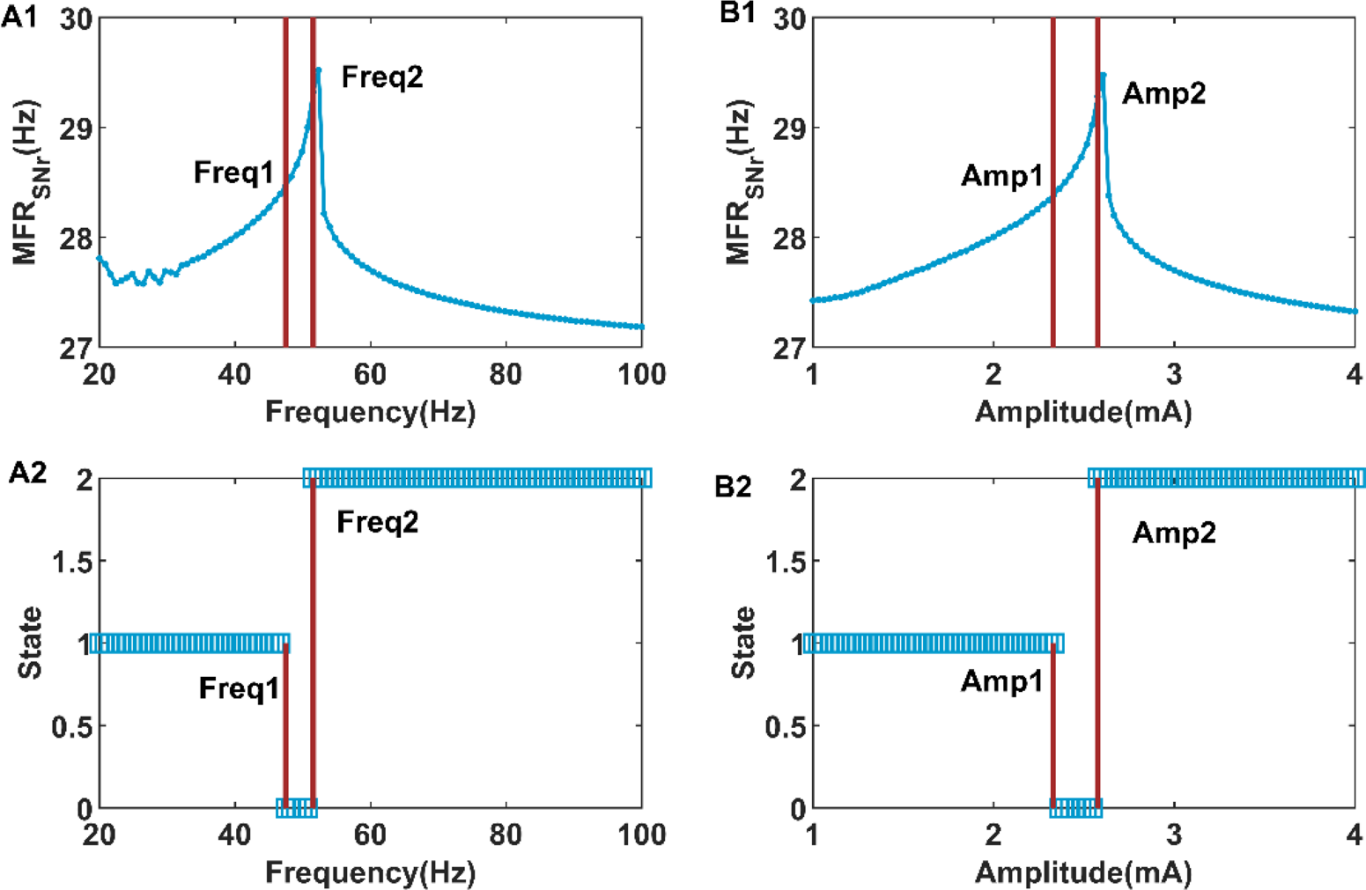
The mean firing rate (**A1**) of SNr and the firing state (**A2**) of cerebral cortex with the increase of DBS stimulation frequency. The mean firing rate (**B1**) of SNr and the firing state (**B2**) of cerebral cortex with the increase of DBS stimulation amplitude. The states 1, 0, and 2 represent SWDs oscillation state, simple oscillation state, and low firing state respectively.

To test whether the above results can be generalized to a larger parameter range, we extended the frequency and amplitude ranges to $\left[ {20,100} \right]$ Hz and $[1,4][1,4]$ mA respectively. As shown in Fig. 9A1, the mean firing rate of SNr increases to a peak at Freq2 and then decreases slowly with increasing frequency. Combined with Fig. 9A1,A2, it is found that the mean firing rate of SNr when the firing activity is in simple oscillation state is higher than that when the firing activity is in SWDs oscillation state. The same situation can be observed in Fig. 9B1, B2. Therefore, it can be concluded that when DBS stimulation intensity increases, including the frequency or amplitude of DBS stimulation, the mean firing rate of SNR increases to a peak at the transition point from simple oscillation state to low discharge state, and then slowly decreases.

### Efficacy of closed-loop control strategy based on frequency modulation

**Figure 10:**
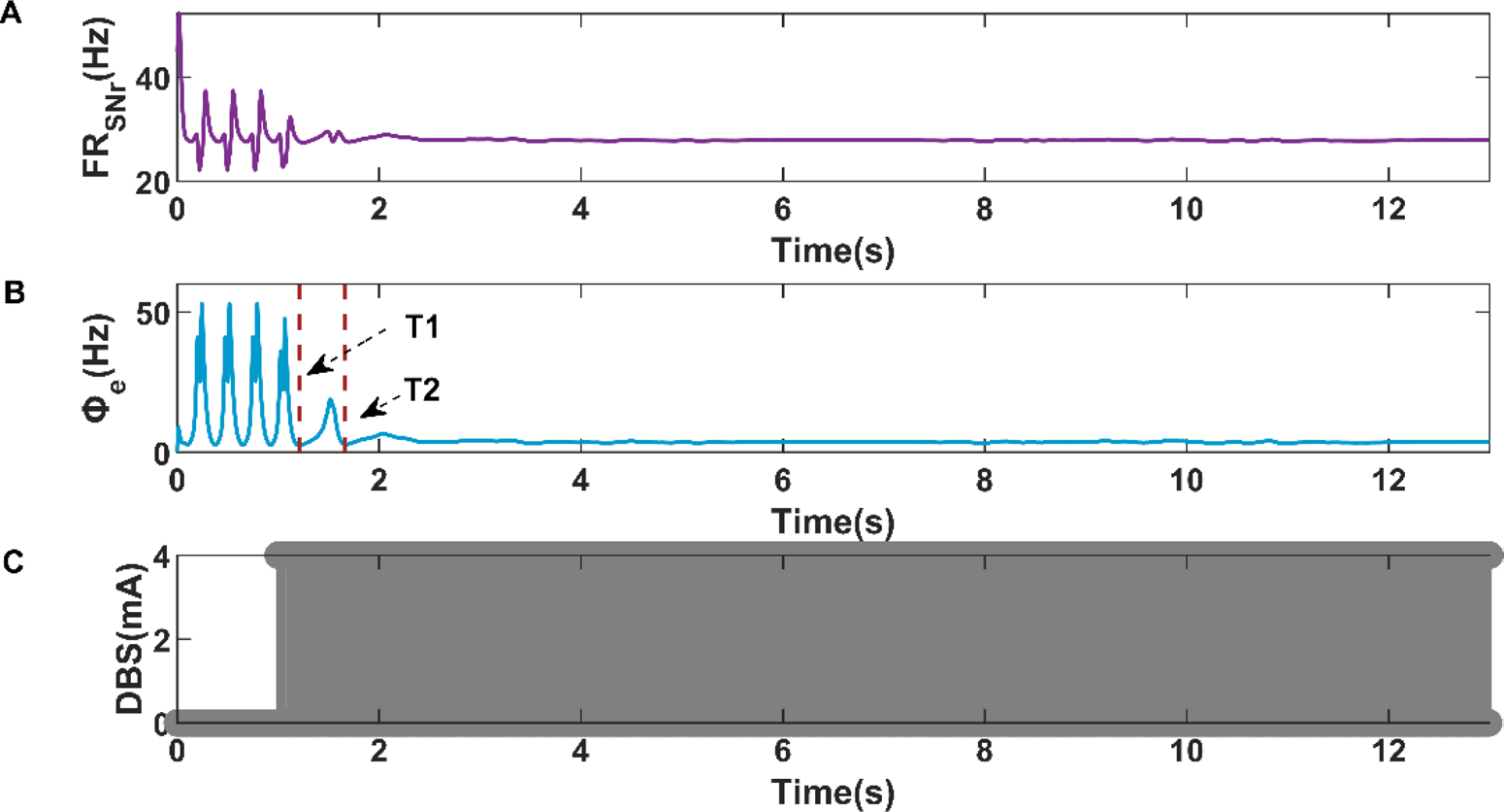
Variations of firing rate of SNr (**A**), firing activity of cerebral cortex (**B**), and the current of closed-loop DBS (**C**) during the closed-loop DBS control when the expected firing rate is 27.7884 Hz.

After the analysis in the previous section, we known that the increase of DBS stimulation intensity will decrease the amplitude of SNr firing rate, which constitutes the theoretical basis for our design of closed-loop DBS controller. In this section, we will focus on the control effect of absence epilepsy under the BoFM strategy. As shown in Fig. 10, we designed a closed-loop controller based on BoFM control strategy for the purpose of controlling the state of cortical discharge activity. Meanwhile, considering the influence of expected firing rate on DBS control results, we selected the mean firing rate of SNr under the condition of cortical low firing state as the expected firing rate, and we applied closed-loop DBS control at 1 s. As shown in Fig. 10B, the cortical firing activity realized the transition from SWDs oscillation state to simple oscillation state at T1 = 1.20825 s, and from simple oscillation state to low firing state at T2 = 1.66255 s. The variations of SNr firing rate and closed-loop DBS current are shown in Fig. 10A,C, respectively.

**Figure 11:**
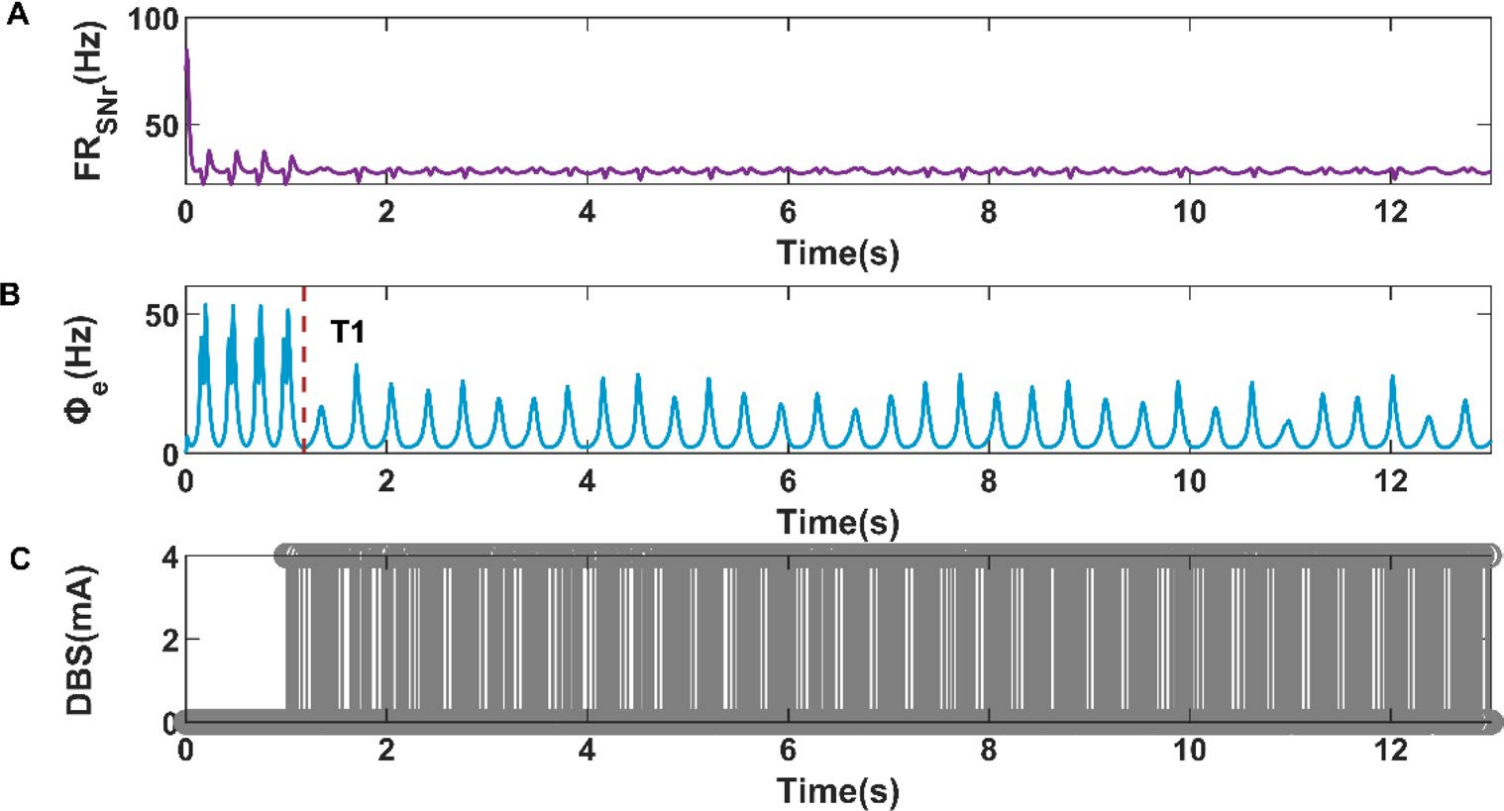
Variations of firing rate of SNr (**A**), firing activity of cerebral cortex (**B**), and the current of closed-loop DBS (**C**) during the closed-loop DBS control when the expected firing rate is 28.4144 Hz.

Moreover, we also selected the mean firing rate of SNr under the condition of cortical simple oscillation state as the expected firing rate to study the control effects of cortical activity. As shown in Fig. 11B, the cortical firing activity realized the transition from SWDs oscillation state to simple oscillation state at T1 = 1.1772s. The variations of SNr firing rate and closed-loop DBS current are shown in Fig. 11A,C, respectively. Compared with Fig. 10B, the cortical firing activity in Fig. 11B did not switch to a low firing state but presented a simple oscillation state. The reason may be that we increase the expected firing rate of the closed-loop controller. Compared with Fig. 11C and Fig. 10C, The DBS current in Fig. 11C is sparser than that in Fig. 10C, which means that the power consumption of the closed-loop DBS current is inversely proportional to the value of the expected firing rate. In addition, an interesting finding is that the response time (T1 = 1.1772s) of simple oscillation in Fig. 11B is smaller than the response time (T1 = 1.20825 s) of simple oscillation in Fig. 10B.

### Efficacy of closed-loop control strategy based on amplitude modulation

In the previous section, we successfully implemented the closed-loop control under the BoFM strategy, and we found that under the BoFM strategy, the control effect induced by different expected rate of fire is different. Considering the influence of DBS amplitude on SNr firing rate, we will focus on the control effect of absence epilepsy under the BoAM strategy in this section.

**Figure 12:**
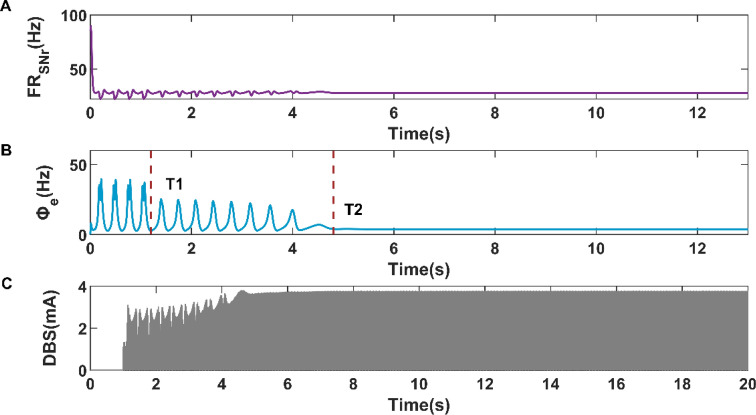
Variations of firing rate of SNr (**A**), firing activity of cerebral cortex (**B**), and the current of closed-loop DBS (**C**) during the closed-loop DBS control when the expected firing rate is 27.7884 Hz.

**Figure 13:**
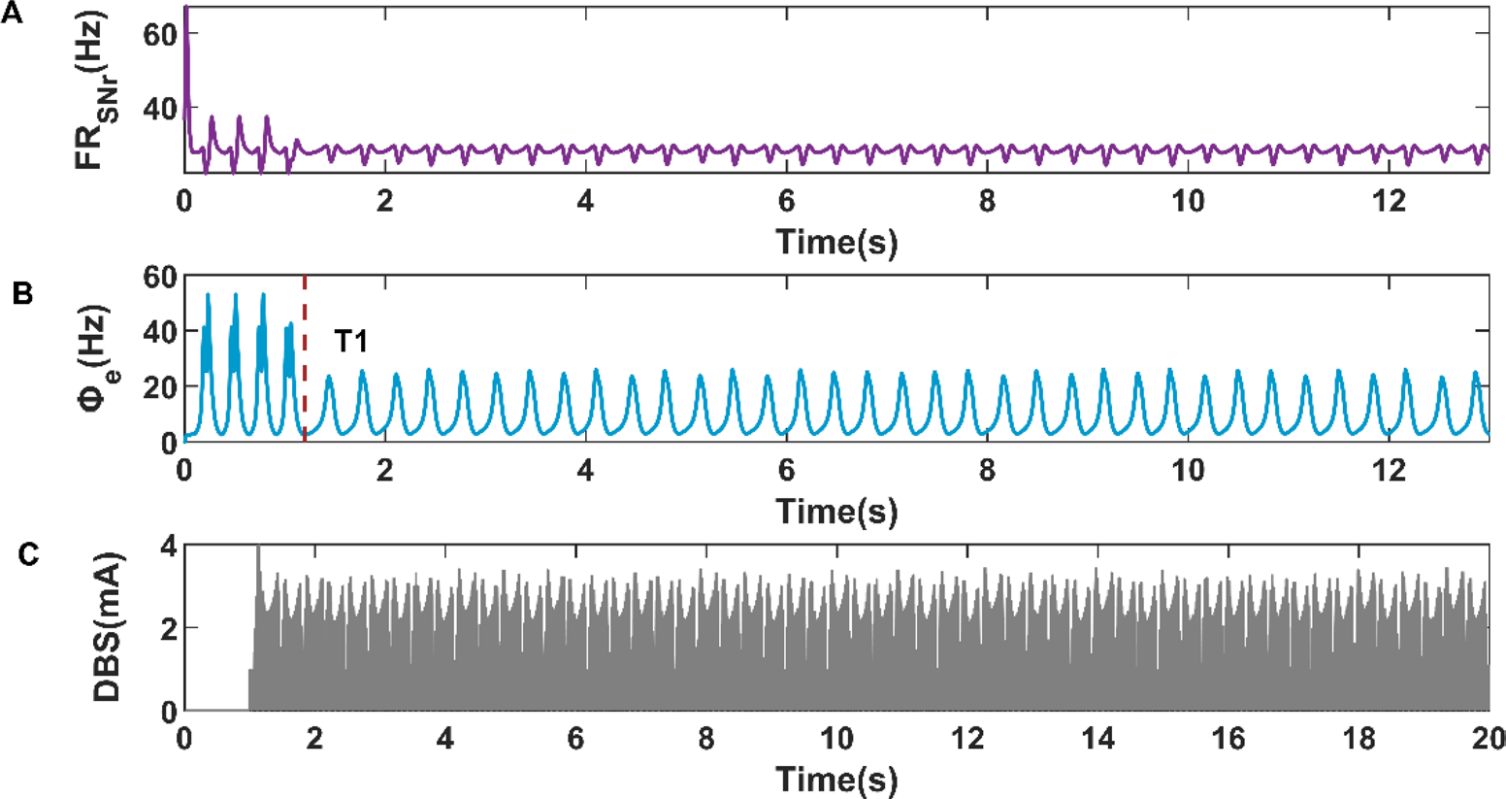
Variations of firing rate of SNr (**A**), firing activity of cerebral cortex (**B**), and the current of closed-loop DBS (**C**) during the closed-loop DBS control when the expected firing rate is 28.4144 Hz.

Like Fig. 10B, when the expected firing rate is 27.7884 Hz, we realize the transition from SWDs oscillation state to low firing state under the BoAM strategy. As shown in Fig. 12B, the simple oscillation state appeared at time T1 = 1.19885 s, and the low firing state appeared at time T2 = 4.8032 s. The variations of SNr firing rate and closed-loop DBS current are shown in Fig. 12A,C, respectively. However, the response time T2 of low discharge state under BoAM strategy is much larger than that under BoFM strategy. Moreover, the amplitude of the closed-loop control BDS current under the BoAM strategy rises from 1 mA to nearly 4 mA and gradually remains stable in Fig. 12C. We also analyze the control of cortical firing activity when the expected firing rate is 28.4144 Hz, which is the mean firing rate of SNr when the cortex is in simple oscillation state. As shown in Fig. 13B, when the expected firing rate is 28.4144 Hz, the cortical firing activity changes from SWDs oscillation state to simple oscillation state at time T1 = 1.19885 s. The variations of SNr firing rate and closed-loop DBS current are shown in Fig. 13A,C, respectively. The response times of simple oscillations are similar in Figs. 12B and 13B, which is different from we concluded in the previous section. In addition, we find that the amplitude of closed-loop DBS current in Fig. 13C is chaotic, which is different from Fig. 12C. However, like the conclusion in the previous section, the DBS current in Fig. 13C is sparser than that in Fig. 12C, which means that the DBS current in Fig. 13C consumes less power than that in Fig. 12C.

## Conclusion and discussion

In this paper, we successfully achieved the conversion of spike and wave discharges (SWDs) oscillating state to other firing state in the cerebral cortex using a closed-loop deep brain stimulation (DBS) controller based on a model of absence epilepsy involving cerebral cortex, thalamus, and basal ganglia. The mean firing rate of substantia nigra pars reticulata (SNr) is selected as a reference signal, and the cortical firing activity is selected as a biomarker reflecting the epileptic state. The controlled auto-regressive (CAR) model and the Routh-Hurwitz stability criterion are introduced to determine the coefficients of the proportional integral (PI) controller. Moreover, we chose the average value of firing rates of simple oscillation and low oscillation respectively as the expected firing rates of the closed-loop DBS controller, which are $r_{rs} = {27}{\text{.7884}}$ Hz and $r_{rs} = {28}{\text{.4144}}$ Hz, respectively.

According to the numerical results, the intensity of the open-loop DBS current, including the amplitude or frequency of the DBS current, will reduce the amplitude of the SNr firing rate. With the increase of current intensity, the mean firing rate of SNr increase firstly and then decrease, and the firing activity of cerebral cortex change from SWDs oscillation state to simple oscillation state and finally kick to low firing state. Therefore, we designed closed-loop controllers based on BoFM strategy and BoAM strategy, respectively. The closed-loop controllers with two control strategies can achieve the desired control effect under different expected firing rates. By adjusting the expected firing rate from 27.7884 Hz to 28.4144 Hz, the final firing state under the closed-loop control is transformed from low firing state to simple oscillation state. Meanwhile, the power consumption of closed-loop DBS current decreases with the increase of expected firing rate. In addition, an interesting phenomenon is that the response time of low firing state under BoAM strategy is much larger than that under BoFM strategy when the expected firing rate is 27.7884 Hz, we hope that the research can provide reference and help for the treatment and prevention of epilepsy patients.

The PI parameters of the closed-loop DBS controller proposed in this paper are calculated by using the stability of the system rather than by traditional trial-and-error adjustment^44^. Therefore, the challenge to apply the proposed closed-loop DBS method to clinical or experimental applications is to identify the relationship between stimulus intensity and reference signal. In our study, the discharge rate of SNr was selected as the reference signal, and the frequency and amplitude of DBS were selected as the stimulus intensity. Although the proposed closed-loop control algorithm can eliminate the SWDs oscillation in the cerebral cortex, some limitations of the study cannot be ignored. First, the PI controller can track the mean firing rate of SNr well only when the change of frequency and amplitude is less than 1 Hz. In other words, a faster change in the target mean firing rate than 1 Hz will lead to an increase in tracking error, which may be caused by unmodeled dynamics. Therefore, to improve the tracking performance of the dynamic reference signal, an adaptive controller is designed to adjust the parameters of PI controller to adapt to the dynamic change of mean firing rate. Second, although TRN has been shown to be effective as a stimulus target for the treatment of absent epilepsy^27,28^, the centromedian thalamic nucleus^59^, subthalamic nucleus^60,61^, and anterior nucleus^62,63^ may also be potential stimulation targets as they are associated with other types of refractory epilepsy. Finally, the basal ganglia-cortical-thalamic network is highly non-linear, so it is necessary to use a non-linear controller in the following work.

## Data Availability

The data used to support the findings of this study are included within the article.
